# Effect of metal oxides on the light radiation intensity of Ba(NO_3_)_2_/Mg-containing pyrotechnic mixtures combustion

**DOI:** 10.1038/s41598-022-22858-x

**Published:** 2022-11-14

**Authors:** Di-hua Ouyang, Chun-hai Yang, Qian-tao Zhang, Hao-yu Yan, Wei-qiang Pang

**Affiliations:** 1 College of Resource Engineering, Xi’an University of Architecture and Technology, Xi’an, 710055 China; 2 Changshu Institute of Technology, Changshu, 215500 China; 3 Xi’an Modern Chemistry Research Institute, Xi’an, 710065 China

**Keywords:** Energy science and technology, Chemical engineering

## Abstract

Studying how to improve the performance of illuminating agents to meet the requirements of ammunition miniaturization of great importance. In this study, a simple method for increasing light radiation intensity through the adding of metal oxides was developed and tested. Results revealed that the metal oxides had a very strong effect on the light radiation intensity of the reaction system. Optical radiation intensity increased by 17.8%, − 5.4% and 25.9% after the addition 5% of MgO, Al_2_O_3_ and BaO to the Ba(NO_3_)_2_/Mg reaction system, respectively. This phenomenon may be related to the light radiation characteristics and reactivity of the metal oxide itself, as well as the temperature at which the added metal oxide can be excited to radiate light intensity.

## Introduction

Pyrotechnics are used in a variety of military applications. Two such of applications are infrared decoy flares and colored signal flares^1–5^. Two ways for changing charge ratios and developing new raw materials have been established to improve combustion efficiency and light radiation intensity, and obtain high color purity in pyrotechnics. Given that the latter method has a long development cycle and costs more than the former method, the former is widely adopted^6,7^. Although Ba(NO_3_)_2_ has a high ignition temperature^8^, it is present in some pyrotechnic flare compositions as an oxidizer, and light producing compositions has been investigated^9–11^. Magnesium is one of the most common fuels because it is inexpensive and easy to ignite. Pyrotechnic formulations containing Mg and Ba(NO_3_)_2_ are used in pyrotechnic illuminating compositions and have been studied in detail^12^. However, early studies on light radiation intensity were generally performed by adjusting the compositions, ratios, and particle sizes of the components under the assumption that optical intensity depends on the energy of the combustion reaction and the reaction rate of the pyrotechnic composition^13,14^. Barkley et al.^15^ explored the effects of microwave illumination on the irradiance and color of Mg/alkali metal nitrate pyrotechnic flames, providing evidence that microwave illumination of alkali-containing pyrotechnic flames may be a useful strategy to achieve dynamic control of light emission intensity. Although this method has a good effect, still, it requires additional microwave light source, high cost and not convenient for practical popularization. Few studies have focused on the effect of metal oxides on the combustion performance^16,17^, especially light radiation intensity of reaction systems.

This study aims to develop a method to improve the light radiation intensity of pyrotechnic mixtures that do not need to change the charge structure and add additional equipment and cost. For this purpose, a simple method for increasing light radiation intensity through the adding of metal oxides was developed and tested. The general idea behind this design is that upon combustion, the metal oxide is heated and then excited to emit light. The observed performances of these new charges showed significant advantages over those of previously known pyrotechnics. In pyrotechnic reaction system, aluminum and magnesium powder are often used as combustible agents, and their corresponding combustion products are aluminum oxide (Al_2_O_3_) and magnesium oxide (MgO), which can emit a lot of strong light. At the same time, barium nitrate is often used as the oxidant of illuminant, and its corresponding decomposition product is barium oxide (BaO). How will they affect the light radiation characteristics of pyrotechnic reaction system? It's not clear yet.

In the present work, MgO, Al_2_O_3_ and BaO were used as additives. The combustion characteristic of Ba(NO_3_)_2_/Mg with them has been studied experimentally using non-contact, far-infrared thermometer and a transient intensity testing instrument. This was done in order to examine the influence of metal oxides on the light radiation intensity of Ba(NO_3_)_2_/Mg-containing pyrotechnic mixtures combustion.

## Experimental

### Materials

The materials used were Ba(NO_3_)_2_, KClO_4_, Mg powder, Al powder, phenolic resin (PR), Al_2_O_3_, MgO and BaO. The purity, particle size and source of the materials are listed in Table 1.

**Table 1:** The purity, particle size and source of the materials.

Material	Purity	Size/mesh	Source
Ba(NO_3_)_2_	pure	90	Anqiu Hongru Chemical Company (Shandong, China)
KClO_4_	pure	90	Richem Company Ltd. (Beijing, China)
Mg/Al	pure	120	Northeast Light Alloy Company Ltd. (Heilong-jiang, China)
PR	pure	90	Shandong Shengquan Chemical Company Ltd. (Shandong, China)
Al_2_O_3_	pure	200	Sinopharm Chemical Reagent Co., Ltd (Shanghai, China)
MgO	pure	200	Sinopharm Chemical Reagent Co., Ltd (Shanghai, China)
BaO	pure	200	Sinopharm Chemical Reagent Co., Ltd (Shanghai, China)

### Preparation of samples

The investigated pyrotechnic mixtures containing Ba(NO_3_)_2_/Mg and KClO_4_/Al with different metal oxides are shown in Table 2.

**Table 2:** Composition of samples by weight.

No	Ba(NO_3_)_2_ [wt %]	KClO_4_ [wt %]	Mg [wt %]	Al [wt %]	PR [wt %]	Al_2_O_3_ [wt %]	MgO [wt %]	BaO [wt %]
1	66	–	31	–	3	–	–	–
2	62		30		3	5	–	–
3	62		30		3	–	5	–
4	62		30		3	–	–	5
5	–	48.5		48.5	3	–	–	–
6	–	46		46	3	5	–	–
7	–	46		46	3	–	–	5

The dry chemicals required to prepare 40 g batches of the formulations presented in Table 2 were weighed out and allowed to dry in an oven overnight at 50 °C. The chemicals were then individually sieved through an 80-mesh screen. The sieved chemicals were mixed with the binder, which was dissolved in acetone before being mixed, and then blended by hand for 20 min. After mixing, the formulations were passed through a 40-mesh sieve. The granules were dried in air for 2–3 h at ambient temperature to ensure partial curing before consolidation. The mixtures were weighed out in two 5 g portions and pressed into pellets by using a manual press and tooling die at a consolidation dead load of 3 MPa with a dwell time of 10 s. The pellets had a diameter of 18 mm, and height of approximately 10 mm. Five pellets of each formulation were pressed and initiated with an electric match at the energy of 10 V.

### Combustion characteristic measurements

**Figure 1:**
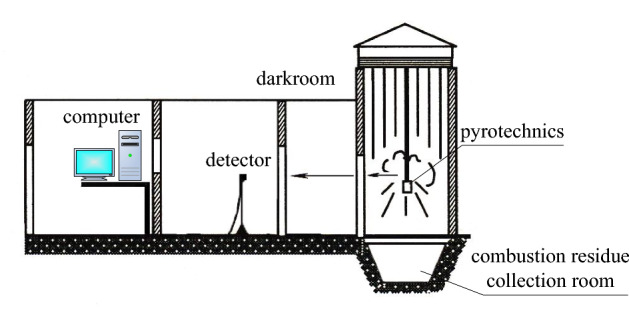
Schematic of the experimental set-up.

Experiments were performed in a laboratory photometric chamber (darkroom). The burning flame temperature of the pyrotechnic mixtures was measured with an IGA-140 non-contact, far-infrared thermometer (The German IMPAC Instrument Company). The spectral range for IGA-140 was 1.45–1.8 µm, and the temperature range was 250–2500 °C. The light radiation intensity of the pyrotechnic mixtures was measured by using a transient intensity testing instrument (Xi’an Institute of Applied Optics) with the spectral range of 390–760 nm and the measuring range of instantaneous light intensity of 10–10^8^ cd. The burn rate of the pyrotechnic mixtures was measured on the basis of target lines at constant pressure. A schematic of the apparatus is shown in Fig. 1. Each set of experiments was repeated five times under the same test conditions, and the mean as given as the experimental results.

## Results and discussion

### Effect of different metal oxides on light radiation intensity

**Figure 2:**
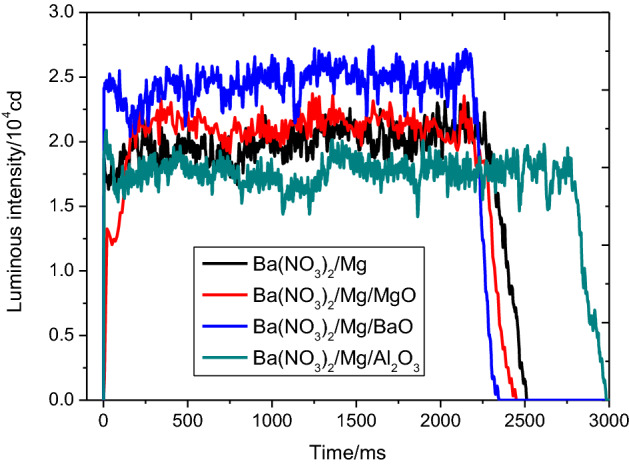
Light radiation intensity-time curves of different metal oxides.

**Figure 3:**
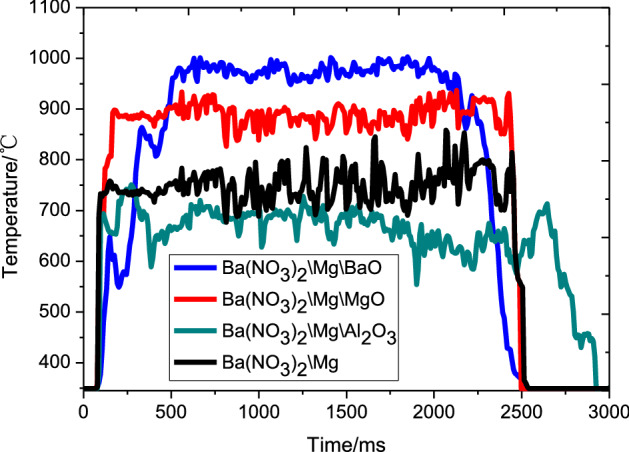
Burning temperature–time curves of different metal oxides.

Figures 2 and 3 display the sets of the light radiation intensity test curves and the burning temperature curves obtained with different metal oxides, respectively. Table 3 shows the average of the five repetitions of experimental results obtained under the same testing condition. As shown in Figs. 2, 3, and Table 3, the burning rate, burning temperature, and light radiation intensity of the Ba(NO_3_)_2_/Mg/Metal oxides, except for those of the Ba (NO_3_)_2_/Mg/Al_2_O_3_ mixtures, were all higher than those of Ba(NO_3_)_2_ /Mg and lower than those of the Ba(NO_3_)_2_/Mg/BaO mixtures.

**Table 3:** Average optical combustion characteristics of Ba(NO_3_)_2_/Mg with different metal oxides.

NO	Average height (mm)	Average burn rate (mm/s)	Average burn temperature ( °C)	Average of temperature relative standard deviation/%	Average light radiation intensity (cd)	Average of light radiation intensity relative standard deviation/%
1	9.30	3.72	752.56	2.1	1.85 × 10^4^	1.6
2	9.70	3.21	668.90	2.2	1.75 × 10^4^	1.8
3	9.43	3.90	889.09	1.6	2.18 × 10^4^	1.4
4	9.34	4.05	969.42	1.8	2.33 × 10^4^	1.6

**Figure 4:**
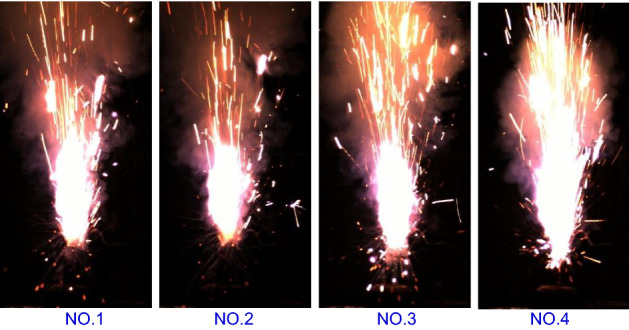
Combustion of the pyrotechnic composition.

Luminescence theory states that, luminescence is caused by electronic motion, and electrons emit the original stored energy during motion. At the macro level, the luminescence of the pyrotechnic reaction system, as shown in the Fig. 4, mainly involved high-temperature particles, liquids and high-pressure gas. The high-temperature particles originated from two components: one was the burning high-temperature particles induced by the gas product, because in general, the increase in combustion speed will lead to an inevitable increase in the content of luminescent particles in the flame per unit of time. And the other was the heated-added metal oxide or the generated high-temperature particle product.

From Table 3, we can know that after the addition of 5% Al_2_O_3_, MgO and BaO to Ba(NO_3_)_2_/Mg, the former reduces the light radiation intensity of the reaction system by 5.6%, and the latter increases the light radiation intensity of the reaction system by 17.8% and 25.9%, respectively. The improvement in light radiation intensity is helpful to the miniaturization of illumination bombs. Small munitions reduce the bulk load to be carried by warfighters. This effect consequently enhances maneuverability and survivability. In particular, the effect of the addition of BaO was the most noticeable.

### Effect of the same metal oxide products on light radiation intensity

On the basis of the reaction equation of the basic system

1 $${\text{Ba(NO}}_{{3}} {)}_{{2}} {\text{ + Mg}} \to {\text{BaO + MgO + 2NO}}_{{2}}$$

We know that the basic reaction system produced BaO when it was reacted. Therefore, we assumed that adding the same metal oxide with the combustion product to the basic reaction system would be conducive to the improvement of light radiation intensity. According to the KClO_4_/Al reaction equation

2 $${\text{3KClO}}_{{4}} {\text{ + 8Al}} \to 3{\text{KCl + 4Al}}_{{2}} {\text{O}}_{{3}}$$

**Figure 5:**
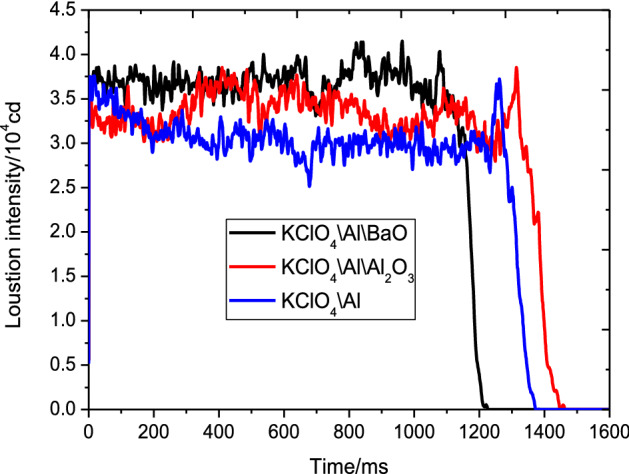
Light radiation intensity-time curve of KClO_4_/Al with different metal oxides.

Al_2_O_3_ was produced after the reaction of the system. BaO and Al_2_O_3_ were added into the KClO_4_/Al reaction system separately to compare their effects on light radiation intensity to verify the above idea. The corresponding results are shown in Fig. 5 and Table 4.

**Table 4:** Combustion characteristics of KClO_4_/Al with different metal oxides.

NO	Average height (mm	Average burning rate (mm/s)	Average temperature( °C)	Average light radiation intensity (cd)	Average of light radiation intensity relative standard deviation/%
5	11.78	8.54	1262.30	3.07 × 10^4^	1.9
6	11.83	8.27	1101.12	3.35 × 10^4^	1.7
7	11.84	9.87	1216.27	3.71 × 10^4^	1.6

However, this result differs from expectation. As shown in Fig. 5 and Table 4, when the KClO_4_/Al reaction system burned, Al_2_O_3_ was produced. However, average light radiation intensity after the addition of Al_2_O_3_ was only 3.35 × 10^4^ cd and had increased by 9.12% relative to the average light radiation intensity the original base formula (KClO_4_\Al). The average light radiation intensity of the react system after the addition of BaO was 3.71 × 10^4^ cd and had increased by 20.85% relative to the average light radiation intensity the original base formula (KClO_4_\Al). That is, the light radiation intensity of the reaction system could be effectively increased by adding a certain amount of metal oxide. However, the metal oxide must not be the same metal oxide as the combustion product.

This phenomenon might be related to the characteristics of the added metal oxide itself. According to the literature^18^, when heated to a certain extent, solid particles (metal oxides) are excited to radiate light energy. Metal oxides have a very strong effect on the light radiation intensity of a reaction system, and high amounts of solid and liquid particles in the flame are associated with high light radiation intensity^18,19^.

The theory of optical radiation was satisfied, and the equation used for the calculation of the light radiation intensity ($I$) of condensed phase particles is as follows^18,19^

3 $$I{ = }\phi_{s} /4\pi = \varepsilon_{D} \cdot A_{s} \cdot \sigma \cdot T^{4} /4\pi$$

here $\sigma$ is the Stephan-Boltzmann constant, which is 5.7 × 10^−12^ W˙cm^−2^˙K^−4^; $\varepsilon_{D}$ is the emissivity of particles (gray body radiation); $A_{s}$ is the flame area formed by combustion; and *T* is the particle temperature. Equation (3) indicates that with the increase in $\varepsilon_{D}$,$\sigma$,$A_{s}$, and *T*, *I* increase under the same conditions. In particular, *T* had the most significant effect.

On the other hand, this result might be attributed to the chemical reaction involving the added BaO during combustion. According to the literature^20^, BaO absorbed the surrounding oxygen for chemical reaction to form BaO_2_ at 450 °C as follows:

4 $$2{\text{BaO}} + {\text{O}}_{2} \mathop{\longrightarrow}\limits^{{450^\circ {\text{C}}}}2{\text{BaO}}_{2}$$

Upon further heating at temperatures above 600 °C, BaO_2_ lost oxygen and reformed BaO as follows:

5 $$2{\text{BaO}}_{2} \mathop{\longrightarrow}\limits^{{ > 600^\circ {\text{C}}}}2{\text{BaO}} + {\text{O}}_{2}$$

During these two chemical reactions, both particles formed and disappeared, and optical radiation was generated outward with a certain intensity. At the same time, neither MgO nor Al_2_O_3_ underwent such a chemical reaction with the surrounding substances (oxygen or Mg powder), and they relied only on their own optical radiation performance as shown in Eq. (1). In particular, Al_2_O_3_ has absorbed some of the energy but might not have been fully excited, and it was removed from the reaction system by the reaction product (gas). Thus, given that Al_2_O_3_ absorbed the energy of the basic reaction system but did not undergo the exothermic reaction and light radiation, the combustion temperature and light radiation intensity of the whole reaction system was decreased (as shown in Figs. 2 and 3). Therefore, the basic formula added with BaO generated stronger optical radiation intensity than the other formulas.

## Conclusions

The combustion characteristic measurements performed in the laboratory photometric chamber (darkroom) showed that strong light radiation intensity could be generated through the addition of metal oxides into the reaction system. This method is simple to operate and has the potential to solve the technical problem of ammunition miniaturization. The following conclusions were drawn:

The metal oxides had a very strong effect on the light radiation intensity of the reaction system. Optical radiation intensity increased by 17.8%, − 5.4%, and 25.9% after the addition of 5% MgO, Al_2_O_3_ and BaO to Ba/Mg reaction system, respectively.

This phenomenon might be related to the optical radiation characteristics and the reactivity of the metal oxide, and the temperature at which the metal oxide was added.

## Data Availability

The datasets generated for this study are available on request to the corresponding author.
